# Oral Presentations 4

**DOI:** 10.1038/sj.bjc.6601930

**Published:** 2004-06-22


### ORAL PRESENTATIONS 4

### 4.1
### MODULATION OF NEURON-RESTRICTIVE SILENCING FACTOR EXPRESSION IN LUNG CANCER

T.A.S Baokbah , C Eccleston , J Chen , J.M. Coulson
Liverpool University, Liverpool, United Kingdom

Small cell lung cancer (SCLC) is characterised by the expression of neuronal genes not seen in non-SCLC (NSCLC) or normal lung. The full-length neuron-restrictive silencer factor (NRSF) is a transcriptional repressor of neuronal genes in non-neuronal cells. We previously identified a splice variant of NRSF that encodes a truncated sNRSF isoform expressed only in SCLC, and aimed to determine its function and downstream targets. We have shown that the expression of several NRSF-regulated genes correlated with that of sNRSF in lung cancer and used reporter constructs based on NRSF-regulated promoters, such as arginine vasopressin (AVP), as readout for sNRSF function. Mutation of an NRSF binding site reduced transcription dependent on the AVP promoter in SCLC by 50% ($p<0.005$), whilst overexpression of sNRSF could activate the AVP promoter in NSCLC where it is normally silenced. Interestingly, overexpression of sNRSF also resulted in significantly increased proliferation of NSCLC ($p<0.05$). RNA interference (RNAi) was used to further investigate the role of sNRSF. Knockdown in lung cancer cells was optimised using published sequences to target other proteins, and five RNAi sequences targeted to NRSF or sNRSF were then designed and evaluated. A SCLC line was established that stably expresses EGFP and sNRSF RNAi, preliminary RT-PCR and immunocytochemistry show down-regulated NRSF. These cells also had a reduced ability to activate an AVP reporter construct. Taken together, these data support the role of sNRSF as a transcriptional activator that antagonises full-length NRSF, and suggest that it may be a key transcriptional regulator in SCLC. We are currently further characterising the full profile of NRSF splicing in SCLC. Lung cancer cells with modulated sNRSF expression will now be used to identify novel target genes regulated by sNRSF in lung cancer by microarray and proteomic analysis. Identification of biological relevant genes will help us to understand the role of sNRSF and may ultimately provide new opportunities for developing detection or treatment strategies.

### 4.2
### STABILITY AND HETEROGENEITY OF EXPRESSION PROFILES IN LUNG CANCER SPECIMENS HARVESTED FOLLOWING SURGICAL RESECTION

FH Blackhall , M Pintilie , DA Wigle , I Jurisica , MS Tsao
Canada, Ontario Cancer Institute, Princess Margaret Hospital and University of Toronto, Toronto

One of the major concerns in microarray profiling studies of clinical samples is the effect of tissue sampling and RNA extraction on the resultant data. We analysed gene expression in lung cancer specimens that were serially harvested from the tumour and snap-frozen at several intervals up to 120 minutes after surgical resection. Global gene expression was profiled on 1.7K cDNA microarrays, and selected stress and hypoxia-activated genes were evaluated using realtime RT-PCR.
Remarkably, similar gene expression profiles were obtained for the majority of samples regardless of the time that had elapsed between resection and freezing. Realtime RT-PCR studies showed significant heterogeneity in the expression levels of stress and hypoxia-activated genes in samples obtained from different areas of a tumour specimen at one time point after resection. The variations between multiple samplings were significantly greater than those of elapsed time between sampling/freezing.
Overall, samples snap-frozen within 30-60 minutes of surgical resection are acceptable for gene expression studies; thus, making sampling and snap-freezing tumour samples in a routine surgical pathology laboratory setting feasible. However, sampling and pooling from multiple sites of each tumour may be necessary for expression profiling studies to overcome the molecular heterogeneity present in tumour specimens.

### 4.3
### MOLECULAR MECHANISMS OF PLATINUM (PT) BASED CHEMOTHERAPY IN NON-SMALL CELL LUNG CANCER PATIENTS (NSCLC) USING MICROARRAYS

RD Petty [1], GI Murray [2], K Kerr [2], MC Nicolson [3], JD Bissett [3], E Collie-Duguid [1]
[1] Department of Medicine and Therapeutics, University of Aberdeen, Aberdeen, United Kingdom, [2] Department of Pathology, University of Aberdeen, Aberdeen, United Kingdom, [3] Department of Oncology, Aberdeen Royal Infirmary, Aberdeen, United Kingdom

**Introduction:** Improved clinical outcomes will result from a better understanding of the molecular mechanisms underlying NSCLC pathogenesis and how these can be exploited by systemic therapies. Cell line studies have suggested that cytotoxic drugs lead to increased mitochondrial membrane permeability (MMP) and that subsequent cell death occurs by caspase independent pathways.
**Methods:** Tumour and uninvolved adjacent paired tissues from NSCLC patients who have received Pt based neoadjuvant chemotherapy have been profiled using Affymetrix HG-U133A GeneChips™). Gene expression data has been validated at mRNA and protein expression levels. Data was analysed using Affymetrix MASv5.0, MicroDBv3.0, DMTv3.0 and Genespring 6.1.
**Results:** In supervised data analysis we have identified contrasting abnormalities in cell death pathways in responding and non-responding tumours. Overall, a comprehensive inhibition of all programmed cell death (PCD) pathways (both caspase dependent and independent) is seen in non-responding tumours, and there is a relative over expression of molecules that decrease MMP. However, in responding tumours while the pathways of caspase dependent PCD are blocked, the pathways of caspase independent PCD appear to be functional, and there is a general down regulation of molecules that decrease MMP and up regulation of molecules that increase MMP. Expression of effector non-caspase proteases involved in apoptosis-like PCD and their inhibitors is strongly correlated with extent of tumour response.
**Conclusions:** These findings suggest that the functionality of caspase independent PCD pathways may be a critical factor in determining response to Pt based therapy in NSCLC patients. Increased MMP may be a key event that may act as a trigger for an apoptotic-like caspase independent PCD. These pathways may provide an important target for novel therapeutics that has not previously been extensively investigated. This approach may be broadly applicable in the optimisation of targeted and conveintional cytotoxic therapies for solid tumours. We are evaluating these pathways in a large series of NSCLC patients using immunohistochemistry.

### 4.4
### A MOLECULAR PROFILE THAT PREDICTS RESPONSE TO PLATINUM BASED CHEMOTHERAPY IN NON-SMALL CELL LUNG CANCER (NSCLC) PATIENTS

RD Petty [1], GI Murray [2], K Kerr [2], MC Nicolson [3], JD Bissett [3], E Collie-Duguid [1]
[1] Department of Medicine and Therapeutics, University of Aberdeen, Aberdeen, United Kingdom, [2] Department of Pathology, University of Aberdeen, Aberdeen, United Kingdom, [3] Departmrnt of Oncology, Aberdeen Royal Infirmary, Aberdeen, United Kingdom

**Introduction:** NSCLC is the most common cause of premature death from malignant disease in western countries. Improved therapy is needed. Optimisation of all systemic therapies would occur if it were possible to predict response. This would facilitate individualisation of treatment planning.
**Methods:** Tumour and uninvolved adjacent paired tissues from NSCLC patients who have received Pt based neoadjuvant chemotherapy have been profiled using Affymetrix HG-U133A GeneChips™). Gene expression data has been validated at mRNA and protein expression levels. Data was analysed using Affymetrix MASv5.0, MicroDBv3.0, DMTv3.0 and Genespring 6.1.
**Results:** Probability (Welch's t-test $p<0.05$) and threshold (>2 fold) filtering identified 2 subset of genes that separated samples into 2 groups based on diagnosis (tumour:normal) or tumour response (WHO criteria) respectively, using unsupervised hierarchal clustering. Response was predicted with 100% accuracy in 'leave one out cross validation' using the KNN algorithm. This gene set has correctly predicted response in an independent tumour sample with high predictive strength (p ratio =0.015) and we are currently profiling more patients to form an independent test set.
**Conclusions:** In this pilot study we have provided a 'proof of principle' that global gene expression profiling can identify a molecular profile capable of predicting response in individual patients with NSCLC. We are currently investigating the ability of the molecular markers identified to predict response to Pt based treatment in pre-therapy specimens from a larger series of patients (approx. 200) with either early (neoadjuvant therapy) or advanced disease (palliative therapy) using IHC. We plan to proceed to prospective studies of global gene expression profiling with the aim of identifying a molecular profile that will allow individualised treatment planning.

© 2004 Cancer Research UK

### 4.5

### PET-BASED MAPPING OF LYMPH NODE SPREAD IN NON-SMALL CELL LUNG CANCER (NSCLC)

DB Landau , S Ahmad , M O'Doherty , T Treasure
Guys & St Thomas' NHS Trust, London, United Kingdom

Introduction: Numerous studies have shown that PET is more accurate than CT at identifying thoracic lymph nodes in NSCLC. The aim of this study is to investigate the pattern of mediastinal nodal involvement in NSCLC in relation to primary tumour location using PET scanning.
Method: Patients were selected from a database of 1400 patients who had PET scans for suspected lung cancer between 2000 and 2002. Tumour position and site of any lymph node metastases were noted. Nodes were considered positive if the Standardised Uptake Value (SUV) was significantly raised relative to the surrounding region.
**Results:** 288 patients out of 513 were node positive on PET. Of 242 upper zone (UZ), 126 midzone (MZ) and 145 lower zone(LZ) tumours, 46%, 40% and 45% respectively were ipsilateral hilar node positive (IHN+), 8%, 5% and 20% respectively were subcarinal node (SCN) positive and 7%, 4% and 2% respectively were tracheobronchial node (TBN) positive. In IHN+ patients 14%, 6% and 23% respectively of UZ, MZ and LZ tumours showed increased activity in the SCN, 11%, 6% and 5% in the TBN and 17%, 7% and 14% in the contralateral hilar nodes (CHN). In IHN negative patients the corresponding figures were 18%, 27% and 70% for SCN, 14%, 18% and 0% for TBN and 21%, 18% and 10% for CHN.
**Conclusions:** IHN involvement is similar regardless of primary tumour location. SCN involvement is more common with LZ tumours. TBN involvement is more common with UZ tumours. The CHN are more commonly involved than other nodal areas more frequently sampled in routine clinical practice. The clinical implication of this finding is that some patients are significantly undertreated. The TBN and SCN groups may represent 2$^{nd}$ station nodes of distinct lymphatic pathways from IH nodes. These results give valuable information for further research into patterns of nodal spread in NSCLC aimed at improving radiotherapy and surgical planning.

### 4.6

### RESPIRATORY MOTION MODELLING FOR OPTIMISATION OF NON-SMALL CELL LUNG CANCER (NSCLC) RADIOTHERAPY

S Ahmad [1], DB Landau [1], JM Blackall [2], M Miquel [2], DJ Hawkes [2]
[1] Guy's & St. Thomas' NHS Trust, London, United Kingdom, [2] King's College London, London, United Kingdom

**Introduction:** Despite recent improvements in NSCLC treatment, respiratory motion significantly impacts on radiotherapy planning. We are developing a system to model respiratory motion by investigating complex 3D lung motion and deformation in 10 healthy volunteers and patients with lung cancer, to create subject-specific models using a novel MR and CT based imaging and non-rigid registration method.
**Method:** Models were constructed using a voxel-based image registration technique to coregister MR images acquired throughout the breathing cycle. A high-quality reference image, acquired at exhale, was aligned to each of a sequence of scans at positions between exhale and inhale. Two different image acquisition techniques were used: 1. free breathing and 2. at various breath-hold positions between exhale and inhale. Positions of all points on the surface of the lung were plotted against diaphragm position, chosen to represent position in the respiratory cycle. Studies were made of different models to analyse inhale and exhale trajectories of breathing. Data from selected areas in the lung were investigated to demonstrate differential lung movement.
Results: Maximum displacement of 16mm, at maximal inhale was observed when comparing inhale to exhale data. Comparison of two breathing cycles showed 19mm displacement, at mid-cycle position. Disproportionately large displacements of up to 27mm were seen when comparing free breathing to breath-hold models. Diaphragm-adjacent lung was most mobile (42mm displacement) especially in the superior-inferior direction. However, up to 10mm lateral movement also occurred at the lung apex.
**Conclusions:** Our results suggest that inter-cycle variation may be greater than intra-cycle variation and that breath-hold models do not accurately represent lung motion when the subject is breathing freely, as they would be during radiotherapy treatment. We believe that modelling technology has the potential to be used for optimisation of radiation delivery and to further develop 4D radiotherapy planning.

### 4.7

### THE TIMING OF THORACIC IRRADIATION IN LIMITED DISEASE SMALL CELL LUNG CANCER

L. E. James [1], A. Hackshaw [1], S. G. Spiro [1], R. M. Rudd [2], P. Clarke [1], C. Trask [3], N. H. Gower [1], P. G. Harper [1], R. S. Souhami [1]
[1] United Kingdom, University College, London, [2] United Kingdom, St. Bartholomew's Hospital, London, [3] United Kingdom, Southend Hospital, Essex, [4] United Kingdom, Guy's Hospital, London

Uncertainty about the optimal timing of thoracic irradiation (TI) led us to undertake a randomised trial comparing survival in patients given 'early' TI (with the 2$^{nd}$ cycle of chemotherapy) with those given 'late' TI (with the 6$^{th}$ cycle of chemotherapy). Our trial aimed to replicate that of an NCIC study (JCO 1993,Vol 11 (2): 336) in which early TI increased 3-year survival by 10%.
Between January 1993 and January 2002, 325 patients were randomised to receive either early TI or late TI. All patients received chemotherapy, which was given every 21 days for 6 courses and consisted of cyclophosphamide, doxorubicin and vincristine, alternating with cisplatin and etoposide. Thoracic radiotherapy dose was 40Gy in 15 fractions over 3 weeks. Prophylactic cranial radiotherapy, 25Gy in 10 fractions over 2 weeks, was given to responding patients with a negative, post treatment, brain scan.
Survival at 3 years was similar between the two arms; 16% in those who received early TI and 20% in those who received late TI (hazard ratio 1.18, 95% CI 0.93-1.51, $p$=0.18). This is in contrast to the NCIC trial. We therefore looked at other trials on the topic:

| Trial | No. of patients | Difference in median survival (early - late), | % who completed ssigned chemo course | |
|---|---|---|---|---|
| | | months | Early | Late |
| Murray 1993 | 308 | +5.2 (p=0.01) | 83 | 84 |
| Jeremic 1997 | 103 | +8.0 (p=0.05) | Similar in 2 arms | |
| Takada 2002 | 228 | +7.5 (p=0.10) | 86 | 88 |
| | | | | |
| Perry 1987 | 270 | -1.5 (p=0.08) | Lower in early arm | |
| Work 1997 | 200 | -1.5 (p=0.41) | 52 | 72 |
| Current trial | 325 | -1.6 (p=0.18) | 69 | 80 |

Three trials showed that early TI was better and 3 trials showed no difference (or early TI slightly worse). The difference in results seem to be associated with chemotherapy uptake. Early TI may only be better if the assigned chemotherapy regimen is maintained and not reduced.

### 4.8

### GEFITINIB ('IRESSA', ZD1839) IN PATIENTS WITH NON-SMALL CELL LUNG CANCER (NSCLC): THE ROYAL MARSDEN EXPERIENCE

NR Maisey , M Parton , C Harper-Wynne , K Sumpter , S Ashley , T Eisen , M O'Brien
Royal Marsden Hospital, London, United Kingdom

**Background**: Gefitinib, an inhibitor of the intracellular tyrosine kinase domain, has demonstrated useful activity in advanced pre-treated NSCLC (IDEAL I / II). The clinical efficacy of gefitinib was assessed as part of an Expanded Access Programme.
**Methods:** Patients (pts) were treated with gefitinib 250mg/day. Endpoints included overall survival (OS) and time to treatment failure (TTF). Objective and symptom responses (OR, SR) were assessed at 1 month intervals.
**Results:** 141 pts were assessed: median age 65 (35-88); male/female, 81/60; performance status (PS) 0/1/2/3, 1/45/39/16%; advanced disease (IIIB/IV), 123pts; median number of prior chemotherapy regimens, 1 (0-4). 125 pts were evaluable for OR: partial response (PR) 6%; stable disease (SD) 55%; progressive disease (PD) 39%. Median duration of disease control (PR + SD) 13 weeks (95% CI 9-17). 131 pts were evaluable for SR: symptom improvement (SI) 31%; No change (NC) 33%; worsening symptoms (WS) 37%. Median duration of symptom control (SI+NC) 11 weeks (95% CI 9-13). The most common toxicity was diarrhoea (Grade 3: 4.5%; Grade IV: 1%). 1 pt died of suspected pneumonitis. Median OS/TTF 20 weeks (95% CI 13-28) / 8 weeks (95% CI 7-10) respectively. Pts (n=12) with bronchoalveolar adenocarcinoma (BAC) had significantly longer TTF than other histological subtypes (median duration 13 and 8 weeks respectively, p=0.04). OR/SR for pts with BAC was 8.3% / 50% respectively. Pts with PS3 had significantly lower OS / TTF (9 and 5 weeks respectively). OR / SR was 0% / 13.3% respectively. Number of prior chemotherapy regimens did not significantly alter OS / TTF.
**Conclusions**: This series of patients shows similar survival/symptom response to published phase II data. BAC pts appear to benefit more than other subtypes. Despite low toxicity, gefitinib does not benefit those with poor PS but can benefit heavily pre-treated patients, since the number of prior chemotherapy regimens did not influence survival.

© 2004 Cancer Research UK

